# Topical composite hydrogel incorporating human amniotic membrane and spidroin for the treatment of chronic wounds in diabetes mellitus

**DOI:** 10.5599/admet.2822

**Published:** 2025-08-24

**Authors:** Suyarta Efrida Pakpahan, Anggraini Barlian, Arie Wibowo, Indra Wibowo

**Affiliations:** 1 Doctoral Program of Biology, School of Life Sciences and Technology, Institut Teknologi Bandung, Ganesha, 10, Bandung, 40132, Jawa Barat, Indonesia; 2 Faculty of Health, Institut Kesehatan Rajawali, Bandung, 40184, Jawa Barat, Indonesia; 3 Animal Physiology, Development and Biomedical Sciences Research Group, School of Life Sciences and Technology, Institut Teknologi Bandung, Ganesha, 10, Bandung, 40132, Jawa Barat, Indonesia; 4 Scientific Imaging Center, Institut Teknologi Bandung, Ganesha, 10, Bandung, 40132, Jawa Barat, Indonesia; 5 Materials Science and Engineering Research Group, Faculty of Mechanical and Aerospace Engineering, Institut Teknologi Bandung, Ganesha, 10, Bandung, 40132, Jawa Barat, Indonesia

**Keywords:** *Argiope appensa*, chronic diabetic wound, *in vivo*, transforming growth factor beta, wound healing process

## Abstract

**Background and purpose:**

Traditional diabetic chronic skin wound dressings often lack the bioactivity required to promote regeneration in these complex wounds. The use of human amniotic membrane (hAM) has been identified as a promising natural option for diabetic skin wound regeneration, but hAM is susceptible to rejection and release of growth factors, so cells must be decellularized and supplemented with biomaterials such as spidroin, a hydrogel delivery system. This study aims to analyse and evaluate a composite hydrogel combining hAM and spidroin proteins to enhance the healing of diabetic chronic wounds.

**Experimental approach:**

Hydrogels were synthesized and characterized using scanning electron microscope (SEM), attenuated total reflectance Fourier transform infrared spectroscopy, physical tests, gel fraction and swelling ratio. Wound healing studies were performed using alloxan-induced diabetic mice. Full-thickness wounds were created and treated with the hydrogel formulations. Macroscopic progress of wound healing was monitored, and histological analysis was performed to assess reepithelialization, inflammatory response, and collagen deposition.

**Key results:**

The functional groups of hAMD components were identified at the characteristic absorption peaks of 1650 cm^-1^, while spidroin showed a peak at 1530 cm^-1^. In particular, the 10 % composite (hAMD + spidroin) showed significantly faster wound closure compared to the control group and other treatment groups. Histological findings confirmed that the 10 % composite was able to facilitate cell proliferation, reduce inflammation, enhance epithelial regeneration, angiogenesis, fibroblast formation and regular collagen matrix and decrease transforming growth factor beta in the remodelling phase.

**Conclusion:**

Composite (hAMD + spidroin) 10 % showed promising wound healing efficacy in diabetic conditions, indicating its potential as a bioactive wound dressing for chronic diabetes.

## Introduction

Skin injuries resulting from trauma, surgical interventions, or genetic disorders such as diabetic ulcers remain prevalent and can lead to tissue damage or functional impairment. One significant underlying cause is diabetes mellitus (DM) [1], a condition that disrupts angiogenesis across various tissues and may ultimately lead to severe outcomes, including limb amputation [2]. Diabetic wounds commonly develop as complications in individuals with diabetes, primarily due to peripheral neuropathy and vascular dysfunction [3,4].

Infections, chronic inflammatory responses, frequently disturbed immune responses, impaired cell proliferation, reduced fibroblast migration, and decreased angiogenesis are common pathological features observed in diabetic wounds [5,6]. The reduced formation of new blood vessels associated with diabetes creates an imbalance that delays the healing phase of the inflammatory response [7,8]. Hyperglycemia decreases the body's ability to fight infections resulting from tissue damage by inhibiting the activity of immune cells [9]. It also reduces tissue oxygenation due to impaired circulation and microvascular dysfunction. Vascular alterations in individuals with diabetes can further limit leukocyte migration to the wound site, thereby increasing the risk of infection [8]. This imbalance in angiogenesis and fibroblast function may lead to chronic, non-healing wounds or excessive scar formation, including conventional scars, keloids, and hypertrophic scars.

Previous treatments used autografts, allografts, and xenografts, but these can be painful, cause new wounds, patient discomfort, donor scarcity, low vascularization, infectious complications, and require very high costs [10]. Another treatment is ointment [11]. This treatment only heals wounds on the surface and cannot penetrate the deep layers of the wound. The main clinical wound treatments are wound accessories, negative pressure drainage, skin grafts, and surgery [12,13]. However, the limitations of these treatments underscore the urgent need to develop innovative therapies and strategies, such as biomaterial skin tissue engineering that offers hydrogels as a safe alternative to induce tissue growth using extracellular matrix (ECM) and growth factors or biomolecules [14]. The development of treatment is carried out in the form of a topical hydrogel because it can maintain wound moisture and can be applied for several days without frequent replacement, thereby reducing patient trauma and pain [15].

Therefore, it is necessary to develop scaffolds from native or synthetic biomaterials, or a combination of both, that can mimic the extracellular matrix. A suitable scaffold, biocompatible and non-toxic, is easy to handle and produce. Its mechanical properties are consistent with the anatomical location of implantation, and it must be easily degraded after being replaced when tissue regeneration occurs [16]. One approach to medical intervention is to replace damaged tissue with replacement tissue acceptable to the body itself [17].

Human amniotic and chorionic membranes (hAMs) are native tissues that have been explored because they are easy to obtain, widely available, and can be used as scaffolding materials [18]. Several studies have shown that intact hAM can increase immunogenicity and donor rejection, resulting in poor performance. Therefore, decellularization of hAM (hAMD) is necessary, but hAMD has low mechanical stability and donor differentiation, making it difficult to use in some applications [19]. Therefore, hAMD must be modified to adjust its properties, either by adding silk, a biological polymer known as spidroin, to the composite material. Cells are an essential component in tissue engineering. Fibroblast cells play a crucial role in the wound healing process. They can synthesize collagen, have an anti-inflammatory effect, and promote increased angiogenesis. Additionally, they have high strength (500 to 972 MPa), do not trigger an immune response (low immune response), and are biocompatible, non-toxic, and biodegradable. *Argiope appensa* is a specific type of garden spider found in Indonesia. This spider is known to have arginine-glycine-aspartate (RGD), a specific amino acid sequence that can help any cell proliferate and differentiate faster [20]. Therefore, this study aims to analyse and evaluate wound closure treated with hAMD, spidroin, and a combination of hAMD and spidroin hydrogel compared with healthy controls, negative controls (untreated DM), and positive controls (DM-treated Carbopol and commercial) for the potential application of wound closure in DM rat models, macroscopic and microscopic histopathology.

## Experimental

### Materials

2-5 % glutaraldehyde (Sigma-Aldrich), 1 % antibiotic-antimycotic solution (ABAM) (Gibco), alloxan monohydrate (Sigma-Aldrich), *Argiope appensa* (padepokan dayang sumbi), bovine serum albumin solution (Gibco), 0.1 M cacodylate buffer (Sigma-Aldrich), Carbopol (Samiraschem Indonesia), chloroform (Merck), 4',6-diamidino-2-phenylindole (DAPI) (Sigma-Aldrich), deionized water (HACS deionized water), dialysis solution (Merck), ethylenediaminetetraacetic acid (EDTA), entellan (Merck), etil alcohol (ethanol) absolut (Merck), etil alcohol (ethanol) level of 70/80/90 % (Merck), eosin (Sigma-Aldrich), fetal bovine serum (FBS) (Gibco), formic acid (Merck), fuchsin (Merck), glucose sticks (Sigma-Aldrich), glycerin (Pudak scientific), human dermal fibroblast (HDF) cells passage 23, hematoxylin (Sigma-Aldrich), human amniotic membrane (hAM) commercial (AmchoPlast FLO), hydrogel, hexamethyldisilazane (HMDS) (Sigma-aldrich), low glucose Dulbecco's modified eagle medium (DMEM) (Sigma-Aldrich), mice (*Mus musculus*) (SITH, ITB), methyl alcohol (methanol) (Merck), 0.9 % sodium chloride (NaCl) (Merck), 0.5 M sodium hydroxide (NaOH (Merck), 10 % neutral buffered formalin (NBF) (Paraform Indopath AKD), 5 % ammonium chloride (NH_4_Cl) (Sigma-Aldrich), Phosphate Buffered Saline with Tween-20 (PBST) (Gibco), 4 % PBS (phosphate buffer saline) (Gibco), picric acid (Sigma-Aldrich), paraffin blocks (Paraform Indopath AKD), secondary antibody (alexa fluor 647) (Elabscience), *Tenebrio molitor/ Hermetia larvae illucens*, Transforming growth factor beta (TGF-β) primary antibody (Elabscience), trichrome mallory staining (Sigma-Aldrich), triethylamine (Sigma-Aldrich), triton (Sigma-Aldrich), and xylol (Sigma-Aldrich).

### Methods

#### Placenta retrieval procedure

The placenta retrieval procedure followed all applicable ethical protocols. The donor received comprehensive information regarding the purpose, procedures, and potential applications of the placenta prior to collection. The donor was allowed to ask questions and received clear and comprehensible answers. The informed consent form was presented to the donor, who read and signed it voluntarily, without coercion. The placenta was obtained through a noninvasive method that did not compromise the safety or health of the mother or the newborn. The collection was conducted only after the donor provided informed consent.

#### Preparation, decellularization, and storage process of human amniotic membrane

The placenta was placed in a sterile container containing a transport medium consisting of 1× PBS with 4 % ABAM solution. The placenta was then washed using a sterile saline solution containing ABAM under aseptic conditions. The amniotic membrane (hAM) was carefully separated from the chorion and transferred into a sterile PBS solution supplemented with 1 % ABAM. The treatment of the hAM in this study followed the procedure described by [19], with slight modifications in the sterilization step using ultraviolet (UV) light. The sterilized human amniotic membrane decellularized (hAMD) was stored at room temperature until further use.

### Cell culture

The morphology of human dermal fibroblast (HDF) cells on the surface of the hAM was observed using a scanning electron microscope (SEM). DAPI staining was performed to visualize cell nuclei and to evaluate cell viability and proliferation. HDF cells were isolated from primary cultures of human foreskin (prepuce) provided by Seno Medika Clinic. The cells were cultured in Dulbecco's Modified Eagle Medium (DMEM) supplemented with 10 % fetal bovine serum (FBS) and 1 % antibiotic-antimycotic solution. Incubation was performed at 37 °C with 95 % humidity and 5 % carbon dioxide using an Esco incubator. The culture medium was replaced every two to three days to ensure optimal cell growth [20].

### Characterization of human amniotic membrane

For the characterization of hAM, an attenuated total Fourier transform infrared spectroscopy (ATR-FTIR) with an Alpha II FTIR Bruker between 1000 and 4000 cm^−1^ was used. This analysis was essential as it enabled the identification of the functional groups present and provided insights into the structural composition of the decellularized human amniotic membrane (hAMD).

### Spidroin extraction and dialysis

Spidroin was extracted from silk produced by the *Argiope appensa* spider. The spiders were maintained in the toxicology laboratory under controlled conditions. Each spider was placed in a modified cage made of cardboard with a hole at the top, covered with mesh cloth to allow air circulation. The spiders were sprayed with water twice daily to maintain hydration. Feeding was carried out twice a week using *Tenebrio molitor larvae* or *Hermetia illucens larvae* as the food source. A total of 0.2 g of spidroin was collected and dissolved in 20 mL of formic acid using a magnetic stirrer set at 1000 rpm for 4.5 hours. A dialysis solution was prepared using a 1:200 dilution of deionized water. The pH of the solution was adjusted to 8.0. Dialysis was conducted for 72 hours, with the solution being replaced every 3 hours to maintain optimal conditions. After dialysis, the resulting solution was measured using a NanoDrop spectrophotometer (Thermo Scientific™ NanoDrop™ Lite) [20].

### Fabrication and evaluation of hydrogel

The hydrogel was formulated using Carbopol as the primary base, triethylamine as the emulsifying agent, glycerin as a humectant, and deionized water as the solvent. The formulation was divided into nine different categories, and each group was prepared in triplicate. The evaluation process was conducted meticulously and consisted of three main stages: organoleptic testing, homogeneity testing, and pH measurement. These procedures were performed according to previously established methods [21].

### Physical feasibility test and characterization of hydrogel

The physical feasibility test of the hydrogel was divided into three stages: organoleptic testing, homogeneity testing, and pH measurement. The organoleptic test was conducted by visually inspecting the colour, shape, and odour of the hydrogel preparation, followed by systematic documentation of the observed characteristics. The homogeneity test was performed by smearing samples taken from the upper, middle, and lower parts of the hydrogel. Each sample was placed on a transparent glass slide and covered with a cover glass to observe the clarity and detect the presence of any lumps or inconsistencies. The pH analysis was conducted to evaluate the compatibility of the hydrogel’s pH with the skin’s physiological pH range of 4.5 to 6.5. The pH of each hydrogel sample was measured three times to ensure accuracy and reproducibility [21].

### Scanning electron microscopy

The fabricated hydrogel was stored at 4 °C for 24 hours, then stored at -20 °C for 24 hours. It was subsequently stored at -60 °C for 24 hours and finally kept at -80 °C overnight. After the freezing process, the hydrogel was lyophilized using a freeze-drying method to obtain a porous structure. Morphological characterization of the hydrogel was done using scanning electron microscopy (SEM) (Hitachi SU3500).

### Attenuated total reflectance Fourier-transform infrared

The fabricated hydrogel was stored at -80 °C overnight, lyophilized by freeze-drying, and then tested using the Bruker ALPHA II ATR-FTIR instrument [22]. The analysis was performed in the spectral range of 1000 to 4000 cm^-1^ to identify the functional groups and evaluate the chemical structure of the hydrogel. Characteristic absorption peaks were observed and compared to known reference spectra to confirm the presence of specific chemical bonds associated with the polymer components.

### Fraction gel

The dried fabricated hydrogel was weighed to obtain the initial dry weight, then soaked in deionized water and stirred using a shaker at 60 rpm for 1 hour to remove unbound components. After soaking, the hydrogel was stored at -80 °C overnight, dried in a freezer for 24 hours, and reweighed. The gel fraction, % was calculated by Equation (1):



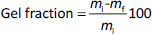

(1)


*m*_i_ : initial dry sample weight, *m*_f_: mixed sample weight

### Swelling ratio

The fabricated hydrogels were immersed in PBS solution and incubated at 37 °C for 24 hours to allow maximum swelling. After incubation, the hydrogels were weighed and stored at -80 °C overnight. The samples were then freeze-dried and reweighed. The swelling ratio was calculated using Equation (2):



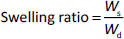

(2)


*W*_s_: weight of the swollen hydrogel, *W*_d_: weight of dry hydrogel

### Alloxan induction and making mice wounds

Alloxan induction and wound creation in mice were conducted with precise methodology. A dose of 120 mg kg^-1^ body weight of alloxan monohydrate was used. The mice were fasted for 16 hours but were still provided with drinking water. They were then randomly grouped, and baseline blood glucose levels were measured using a glucometer. Alloxan monohydrate solution was injected intraperitoneally with exact precision. After injection, the mice were given standard food and water. A stabilization period of six days followed the induction, after which blood glucose levels were measured again. Mice with blood glucose levels of 200 mg dL^-1^ or higher were considered to have developed hyperglycemia [23]. The experiment then proceeded to the wound creation stage, which was performed meticulously. The mice were anesthetized using chloroform. The shaved skin area was disinfected using 70 % alcohol. A skin incision measuring 1×1 cm was made until it reached the dermis, as indicated by the presence of bleeding [24,25]. Wound treatment was subsequently administered by applying the hydrogel formulation topically to the DM mice three times a day.

### Skin tissue collection and histopathological preparation

Before the mice were euthanized on the designated days (days 3, 7, 14, and 21), the wounds were measured macroscopically using a caliper. After the treatment period, the skin tissues were carefully collected and processed for hematoxylin-eosin (HE) staining. The epidermal tissue was placed into tubes containing 10 % neutral buffered formalin (NBF) and fixed for 24 hours. Each tissue sample was then inserted into a 1×1 cm embedding cassette [26]. The samples underwent a dehydration process using graded ethanol concentrations of 70, 80 and 90 %, followed by a clearing process using xylol I and xylol II. The cleared tissues were then infiltrated with molten paraffin using a tissue embedding machine. Once the paraffin began to solidify, it was returned to the embedding machine to complete the infiltration process, after which the tissues were left to solidify fully. The paraffin blocks were sectioned into 3.5 μm-thick slices using a microtome. The tissue sections were mounted onto glass slides and dried overnight in an incubator at 60 °C. Finally, the dried sections were ready for general HE stains [27].

### Hematoxylin-eosin staining

Hematoxylin-eosin staining was a pivotal technique used in histology to enhance the visualization of tissue structures under a microscope. The staining process began with deparaffinization by immersing the slides sequentially in xylol I, xylol II, and xylol III. Rehydration was performed using graded alcohol solutions with decreasing concentrations from absolute ethanol to 70 %. The slides were then rinsed with distilled water to remove any residual alcohol. Following rehydration, the slides were incubated in hematoxylin solution for 10 minutes. After staining, the slides were rinsed under running tap water for 15 minutes to develop the nuclear (hematoxylin) stain. Subsequently, the slides were immersed in eosin solution for 5 minutes to stain the cytoplasmic components. Dehydration was performed by immersing the slides for 3 minutes each in alcohol solutions of 70, 80 and 90 %, and absolute ethanol. Finally, the stained slides were mounted with Entellan mounting medium to preserve the tissue sections and prepare them for microscopic examination [28].

### Mallory trichrome staining

Mallory trichrome staining was used to observe collagen density in tissue sections. Trichrome Mallory staining was used to observe collagen density. The slides were sequentially immersed in xylol I, xylol II, and xylol III for 10 min each. They were then rehydrated through a graded series of alcohols: absolute alcohol, 95 % alcohol and 70 % alcohol with 10 dips in each solution. After rehydration, the slides were immersed in distilled water for 1 minute. The slides were then soaked in picric acid that had been preheated in an oven and subsequently cooled in cold water. After cooling, the slides were stained with Weigert’s hematoxylin for 10 to 15 minutes and rinsed under running water. The slides were immersed in hot water for 10 minutes and then cooled in cold water. Following this, the slides were stained with acid fuchsin for 5 minutes and rinsed again with distilled water. Subsequently, the slides were immersed in phosphotungstic acid for 20 minutes without a rinsing step. They were then stained with methyl blue for 20 minutes and rinsed with water. Dehydration was carried out using increasing concentrations of alcohol 70 and 95 %, and absolute ethanol, with 10 dips in each. The slides were then oven-dried and immersed in xylol solution. Finally, the stained slides were mounted using a coverslip for microscopic observation [29].

### Immunohistochemical staining

The paraffin blocks were sectioned using a 2.5 μm microtome. The ribbon-shaped sections were spread over warm water at 40 to 45 °C and were immediately collected using glass slides. The slides were placed on a hot plate and heated for ten minutes at 60 °C. The prepared slides were immersed in xylol twice, each for ten minutes, to remove paraffin. They were then washed twice with serial ethanol concentrations, starting with 100 % ethanol for ten minutes each. The slides were further rinsed sequentially in 95, 70 and 50 % ethanol, each for five minutes, followed by a final rinse with deionized water for five minutes. The slides were rehydrated with PBS-Triton (PBS 1× + 1 % Triton X-100) for ten minutes. Excess PBS was removed using tissue paper. A 1 % BSA solution was added to the slides to block nonspecific binding, and the slides were incubated at room temperature for 30 minutes. The BSA solution was then discarded. The primary antibody for TGF-β was diluted at 1:400 and applied to the slides, which were incubated overnight at 4 °C. After incubation, the slides were washed three times with PBS-Triton, each for fifteen minutes. A secondary antibody (Alexa Fluor 647), diluted at 1:100, was added and incubated in the dark at room temperature for 30 to 60 minutes. The slides were then washed five times with PBS-Triton, each for fifteen minutes. DAPI (300 μL) was applied to the slides and incubated in the dark at room temperature for 3–5 minutes, followed by a final rinse with PBS-Triton for 1.5 hours. The preparations were mounted with glycerine and covered with a cover glass. The edges of the preparations were sealed and mounted with Entellan [30].

### Data analysis

Statistical analysis was performed using GraphPad Prism 7 for Windows (GraphPad Software, Inc.) (www.graphpad.com). The collected data were analysed using a one-way analysis of variance (One-Way ANOVA) to assess differences among groups. For non-parametric data, the Mann-Whitney U-test was applied to determine the significance of differences between groups. A *p*-value of less than 0.05 was considered statistically significant.

## Results and discussion

### Characterization of human amniotic membrane decellularized

**Figure 1.**
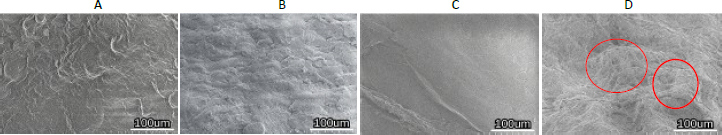
Morphology of hAM: (A) fresh hAM without cells; (B) fresh hAM (hAMF) with cells; (C) Decellularized hAM without cells; (D) Decellularized hAM with remaining cells. Fibroblast cells are indicated by red circles

The characterization results can be seen in Figure 1B, which shows the SEM results of fresh hAM used with cells. The results reveal a substantial presence of cell accumulation or clustering due to the persistence of epithelial cells on the hAM substrate. Figure 1D illustrates the attachment of fibroblast cells to the surfaces of all scaffold materials. The SEM data indicate the presence of fibroblast cells, as marked by a red circle.

**Figure 2.**
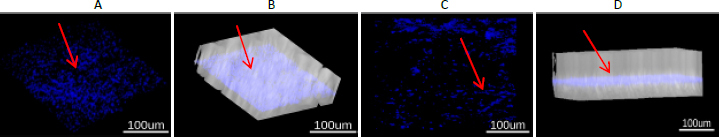
DAPI staining and 3D visualization of fresh and decellularized hAM. A. 3D visualization of HAMF. B. Side and top views of hAMF; C. 3D visualization of HAMD; D. Side and top views of hAMD. Red arrows indicate human dermal fibroblast (HDF) cells grown on hAMF and hAMD

Confocal microscopy was used to visualize the presence of cell nuclei in hAMD and hAMF with DAPI labelling. In Figures 2A and 2C, the freshly seeded hAM exhibits a high cellular density, suggesting the potential overlap between the epithelial cells derived from the HAMF and the seeded HDF cells. In contrast, the hAMD displays a uniform distribution of cells without any noticeable accumulation. The data presented in Figures 2B and 2D depict the 3D DAPI staining observed from both the top and side perspectives. The cell's HDF is positioned centrally.

**Figure 3.**
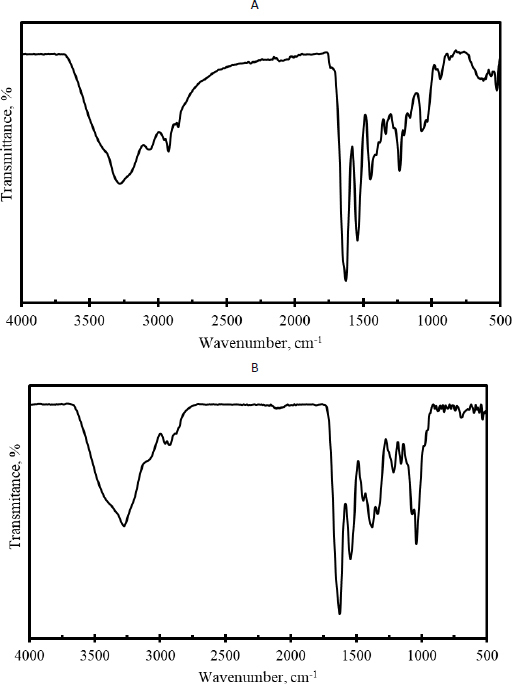
ATR-FTIR Spectra of decellularized hAMD and Spidroin extracted from *Argiope appensa*: (A) Decellularized hAM exhibited characteristic absorption peaks at 1652 cm^-1^ corresponding to C=O stretching vibrations of amide compounds, 1550 cm^-1^ attributed to C=N bonds (from amine or amide groups), and 1339 cm^-1^ associated with S=O bonds (sulfur-oxygen). (B) Spidroin from *Argiope appensa* showed a prominent absorption band at 1530 cm^-1^, indicative of amide group II. These spectra were obtained following spidroin protein extraction and dialysis

The ATR-FTIR analysis of decellularized hAMD at specific wavenumbers, such as 1652, 1550, 1339 and 1200 cm^-1^ (Figure 3A) and the ATR-FTIR analysis of spidroin *Argiope appensa at* wavenumber 1530 cm^-1^.

The results of protein extraction and dialysis (Table 1) indicate that the spidroin protein content was higher in the spider web (net) sample compared to the cocoon, with values of 6.25 ± 0.09 and 3.09 ± 0.05, respectively. This study utilized spidroin protein extracted from the spider net.

**Table 1.** Spidroin protein test results

Spidroin source	Content of spidroin protein, mg
Net	6.25±0.09
Cocoon	3.09±0.05

The ATR-FTIR analysis of spidroin *Argiope appensa* in material characterization reveals the presence of functional groups associated with hAM at a specific wavenumber of 1530 cm^-1^ (Figure 3B).

### Physical feasibility test and characterization of hydrogel

Organoleptic results of 9 types of hydrogels; Carbopol, commercial 10 % (AmchoPlast FLO), commercial 15 % (AmchoPlast FLO), hAMD 10 %, hAMD 15 %, spidroin 10 %, spidroin 15 %, composite 10 % (hAMD + spidroin), composite 15 % (hAMD + spidroin) showed organoleptic properties according to the established specifications, which include transparency, odorless, and flexible and soft texture. For 21 days, no odor, color, or texture changes were observed in all hydrogels.

The pH results for Carbopol, commercial hAMD, hAMD, spidroin, and composite (hAMD + spidroin) have met the specifications for the skin pH range 4.5 to 6.5. All hydrogels comply with the specifications because the resulting hydrogels have a pH value that is not too acidic. All hydrogels from days 0 to 21 were still in a homogeneous condition. The optimal preparation formula meets the hydrogel evaluation requirements and can maintain stability during 21 days of storage at 4 °C, even though the formula does not use preservatives.

### Characterization of hydrogels

**Figure 4.**
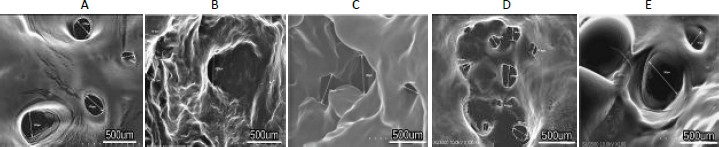
The morphologies of the hydrogels: A - Carbopol; B - Commercial 15 %; C - hAMD 15 %; D - Spidroin 15 % and E - composite 10 %. Arrows are the size of the hydrogel pores

Characterization tests were conducted on hydrogels fabricated with several materials. Figure 4 shows the morphologies of the hydrogels. Pore size consideration in hydrogels is crucial for adjusting their properties. Adjusting the molecular weight of the polymer through hydrolysis enables the manipulation of pore size.

**Figure 5.**
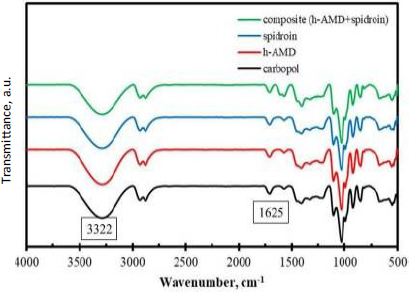
ATR-FTIR characterization of hydrogel: Carbopol (black); hAMD (red); spidroin (blue); composite (hAMD+Spidroin) (green)

The ATR-FTIR analysis of the hydrogel confirmed the presence of various functional groups relevant to its composite materials (Figure 5). The absorption band of Carbopol hydrogel appeared at a wavenumber 3322 cm^-1^, which corresponded to N–H or O–H stretching vibrations (blue colour). This band typically originated from protein-based components such as collagen and spidroin, as well as from hydrogen bonding involving bound water. Its presence indicated that the amniotic membrane retained structural proteins essential for cell proliferation and tissue regeneration.

The absorption band of hAMD was observed at approximately 1625 cm^-1^ (yellow colour), which is typically attributed to the amide I band. This band is derived from C=O stretching vibrations in protein backbones, confirming the presence of collagen, a key protein involved in tissue repair and wound healing. The absorption band of spidroin was detected at a wave number of 1530 cm^-1^ (green colour), representing the amide II band, which is associated with N–H bending and C–N stretching vibrations. This confirmed the incorporation of spidroin, a protein known for its biocompatibility, mechanical strength, and regenerative potential.

**Figure 6.**
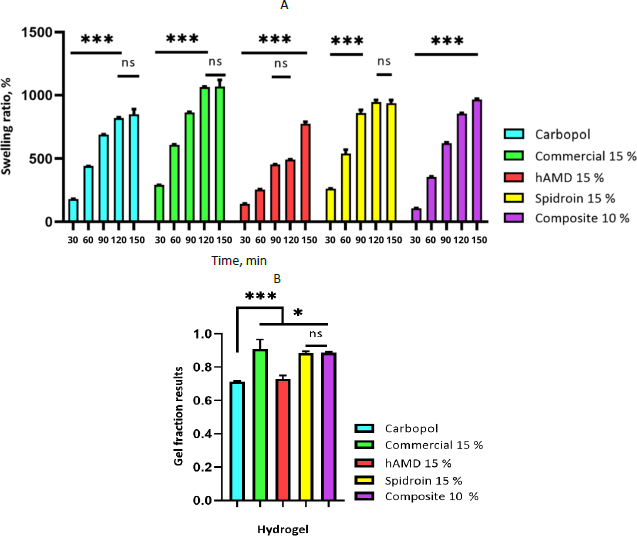
A - swelling ratio of hydrogel, B - gel fraction of hydrogel. The symbol *** indicates a significant difference (*p*<0.05) *n* = 3

The hydrogel fractions are shown in Figure 6B. Each hydrogel formulation has a slight change in the sol fraction. Swelling studies indicated that equilibrium swelling of the hydrogel had been reached after being immersed in PBS overnight. The results showed that the swelling ratio increased with time (Figure 6A).

### Mice wound closure

In a preliminary test, concentration was determined on healthy and DM rats that were not treated and treated with concentrations of 10 % and 15 %. On the 21^st^ day, the wound had closed in the healthy and DM-treated rats, but in the DM rat group, the wound had not closed.

**Figure 7.**
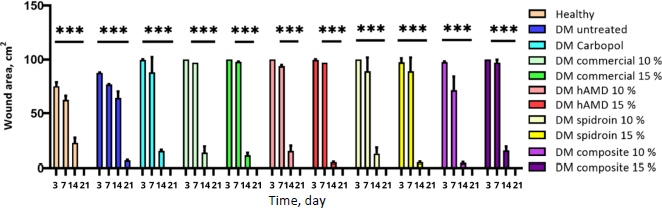
Preliminary test statistical data determines the fastest concentration in wound closure (macroscopic). From the picture above, the quickest wound closure based on concentration includes commercial 15 %, HAMD 15 %, spidroin 15 %, and composite 10 %. The symbol *** indicates a significant difference (*p*<0.05), *n* = 2

On the third day after treatment of nine healthy groups, untreated DM and with various concentrations namely Carbopol (control), commercial 10 and 15 %, hAMD 10 and 15 %, spidroin 10 and 15 %, composite 10 % (hAMD 10 % + spidroin 10 %), and composite 15 % (hAMD 15 % + spidroin 15 %)—wound closure had occurred in the healthy, untreated DM and spidroin 15 % and composite 10 % groups. On day 7, wound closure was observed in all groups; however, only the healthy, untreated DM, spidroin 10 %, spidroin 15 %, and composite 10 % groups showed statistically significant results. On day 14, almost all treatment groups showed significantly higher wound closure scores (*p* < 0.05) compared to the Carbopol control group, except for the composite 15 % group. When comparing the commercial (10 and 15 %), hAMD (10 and 15 %), and spidroin (10 and 15 %) treatment groups, wound closure was 15 % higher in all 15 % concentration groups. Meanwhile, in the composite group (10 and 15 %), wound closure was 10 % higher than in the control (Figure 7).

**Figure 8.**
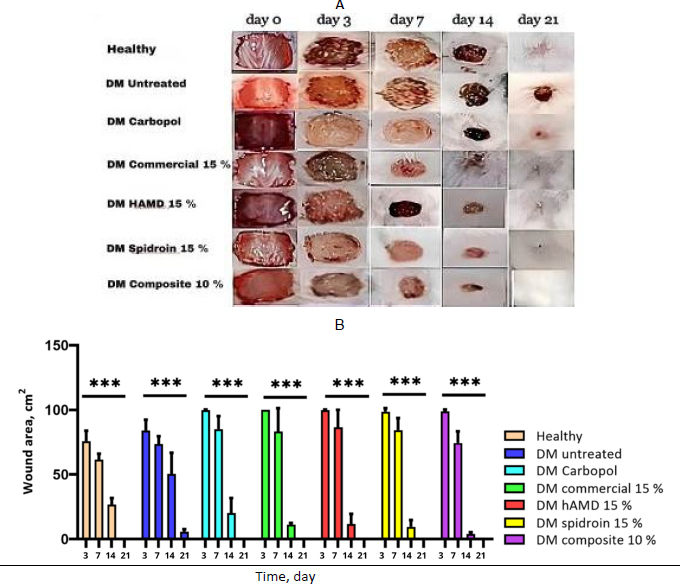
A - wound closure (macroscopic); healthy and DM (untreated), DM treated five groups and B - the quantification of wound closure. The symbol *** indicates a significant difference (*p*<0.05), *n* = 4. Histopathology results using HE staining on days 3, 7, 14 and 21 after the hydrogel treatment

The results of macroscopic observation revealed that, in the untreated group of healthy diabetic rats, wound closure began at the edge on day 3 (Figure 8A).

All treated groups also showed signs of wound closure on day 3, although the changes were not statistically significant (ns). On day 7, wound closure was observed in all treatment groups, as indicated by statistical significance (**). By day 14, the wounds had dried in all treated groups, and a significant difference in wound closure was found between the Carbopol hydrogel and both the 15 % spidroin hydrogel and the 10 % composite group (***), except in the healthy group and the untreated diabetes mellitus (DM) group. The best wound closure was observed in the 10 % composite group. By day 21, complete wound healing had occurred in all treated groups, except in the DM group, which showed no complete closure (Figure 8B).

**Figure 9.**
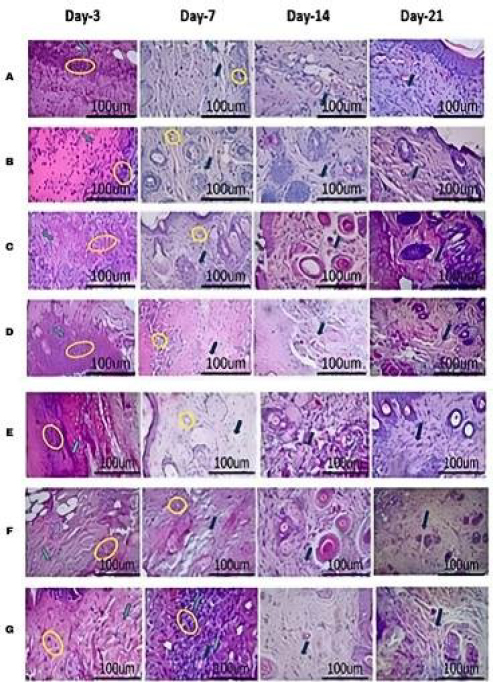
Histopathological results of HE staining on days 3, 7, 14, and 21 after wound creation: (A) healthy untreated, (B) DM untreated, (C) DM treated Carbopol, (D) DM treated commercial 15 %, (E) DM treated hAMD 15 % (F) DM treated spidroin 15%, (G) DM treated composite 10 %. Neutrophils (yellow circles), macrophages (black arrows), lymphocytes (green arrows)

**Figure 10.**
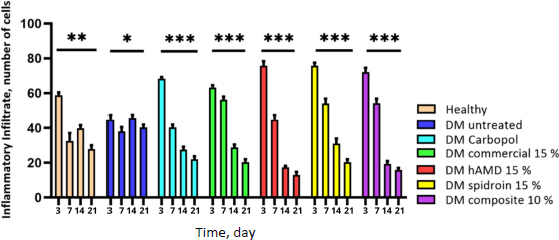
The histogram results showed an increase in the number of inflammatory cells on day 3 for all groups; on day 7, all groups experienced a decrease in the number of cells except for the healthy group and untreated DM; on day 14, all groups experienced a decline except for the healthy group and untreated DM, and on day 21 all groups experienced a decrease in the number of inflammatory cells. The symbol *** indicates a significant difference (*p*<0.05), *n* = 4

All groups reached the maximum number of inflammatory cells, namely neutrophils, macrophages and lymphocytes (Figure 9) at the inflammatory stage or day 3. At the proliferation stage or day 7, inflammatory cells in all groups decreased significantly (***) (Figure 10), but the untreated DM group decreased significantly (*); on day 14, all treated DM groups experienced a sharp decrease in inflammatory cells, but the healthy group and untreated DM increased again. At the remodelling stage on day 21, there was a decline again in all healthy groups, the untreated DM group and all treated groups.

The amount of angiogenesis increased in all groups from day 3 to day 7, indicating a positive response to the treatments. However, on day 14, it decreased in all groups except the commercial group, suggesting a potential limitation of these treatments. On day 21 (Figure 1, histology; Figure 12, histogram), all groups saw a reduction in the amount of angiogenesis except the DM group, untreated, highlighting the potential of this group for further investigation.

**Figure 11.**
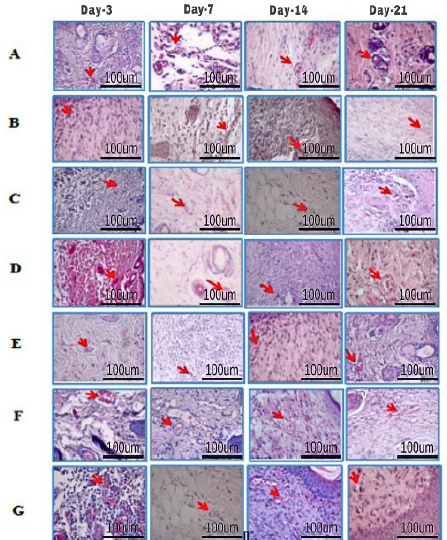
Histological images of mouse skin tissue with HE staining on days 3, 7, 14, and 21. Our comprehensive study of angiogenesis (red arrow) and its role in supplying oxygen and nutrients to the wound site is essential. A - healthy untreated, B - DM untreated, C - DM treated with Carbopol, D - DM treated with commercial 15 %, E - DM treated with hAMD 15 %, F - DM treated with spidroin 15 % and G - DM treated with composite 10 %. Angiogenesis cells in the DM group were treated with 10 % more composite than the healthy group, untreated DM negative control group, and other treatment groups

**Figure 12.**
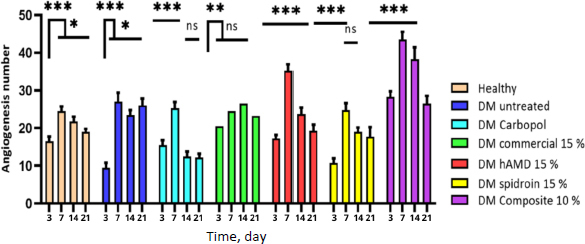
Number of angiogenesis histogram: there was an increase in the amount in all groups from day 3 to day 7. However, on day 14, there was a decrease in all groups except the commercial group, and on day 21, all groups experienced a reduction in the amount of angiogenesis except the untreated DM group. Symbol *** indicates a significant difference (*p*<0.05), *n* = 4.

**Figure 13.**
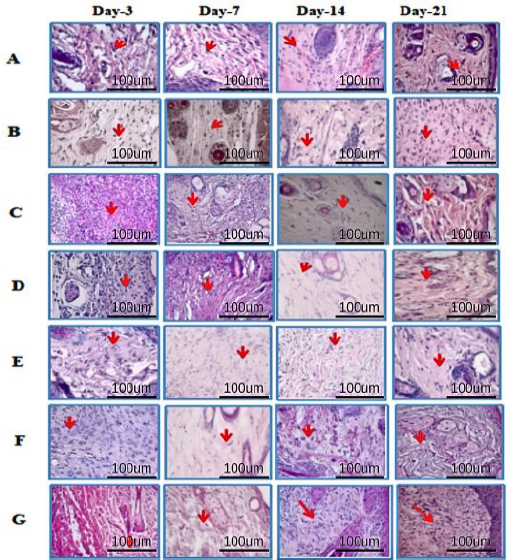
Histological images of mouse skin tissue with HE staining on days 3, 7, 14, and 21. Fibroblasts (red arrows) are essential for forming new ECM and collagen structures. Our findings on fibroblast activity in different treatment groups, including A -healthy untreated, B - DM untreated, C - DM treated with Carbopol, D - DM treated with commercial 15 %, E - DM treated with hAMD 15 %, F - DM treated with spidroin 15 % and G - DM treated with composite 10 %, could potentially revolutionize future treatments. Fibroblast cells in the DM group were untreated with 10 % more composite than the healthy group, untreated DM negative control group, and other treatment groups

**Figure 14.**
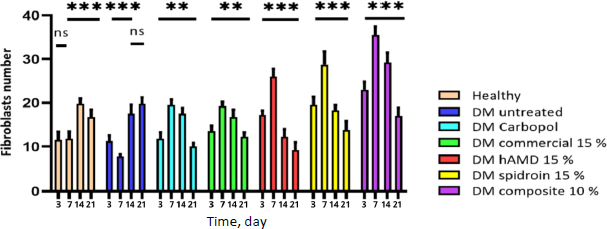
Number of fibroblasts histogram: there was an increase in the number in all groups from day 3 to day 7, except the untreated DM group. However, on day 14, there was a decrease in all groups except the healthy group and untreated DM. On day 21, all groups experienced fewer fibroblasts except the DM group, which was not treated. Symbol *** indicates a significant difference (*p*<0.05), *n* = 4.

On days 3 and 7, the number of fibroblasts significantly (***) increased in all treatment groups, particularly in the 10% composite group (Figure 13, histology; Figure 14, histogram). This increase was likely due to myofibroblast activity, which contributed to accelerated wound healing.

**Figure 15.**
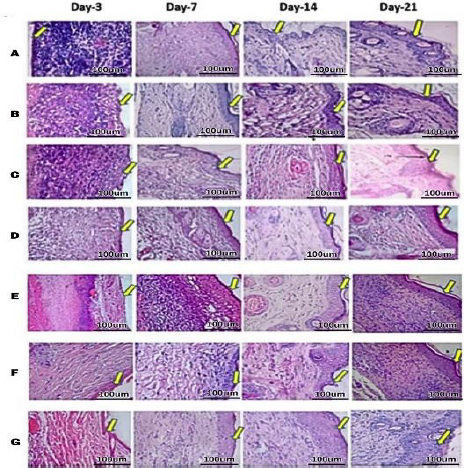
The effect of hydrogel treatment on the formation of reepithelialization (yellow arrow) using HE staining on days 3, 7, 14, and 21. Reepithelialization is in the dermal layer of the skin. A - healthy, B - DM untreated, C - DM treated with Carbopol, D - DM treated with commercial 15%, E - DM treated with hAMD 15%, F - DM treated with spidroin 15% and G - DM treated with composite 10%

**Figure 16.**
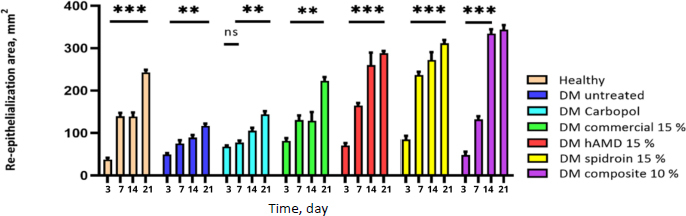
Results of the histogram of the amount of re-epithelialization showed there was an increase in the thickness of re-epithelialization in all groups from day 3 to day 7; on day 14, there was an increase again in all groups except the DM group without treatment; on day 7 to day 14, and there was a decrease again in all groups except the healthy group without treatment. From day 14 to day 21, there was an increase except for the 10 % composite group. The symbol *** indicates a significant difference (*p*<0.05), *n* = 4.

On days 14 and 21, there was a decrease, but in the untreated DM group, there was an increase again. These findings suggest potential future treatments that could harness the power of myofibroblast activity for accelerated wound healing. The histopathological results of re-epithelialization (Figure 15, histology; Figure 16, histogram) and histogram revealed a significant increase (***) in all groups from days 3 to 21.

This substantial increase underscores the crucial role of our research in understanding the dynamics of re-epithelialization. However, in the 10 % composite-treated DM group, on day 21, the rise in re-epithelialization thickening was not significant (ns). The untreated DM group increased from day 3 to day 21, increasing ns, possibly due to factors such as impaired keratinocyte function and inflammation.

**Figure 17.**
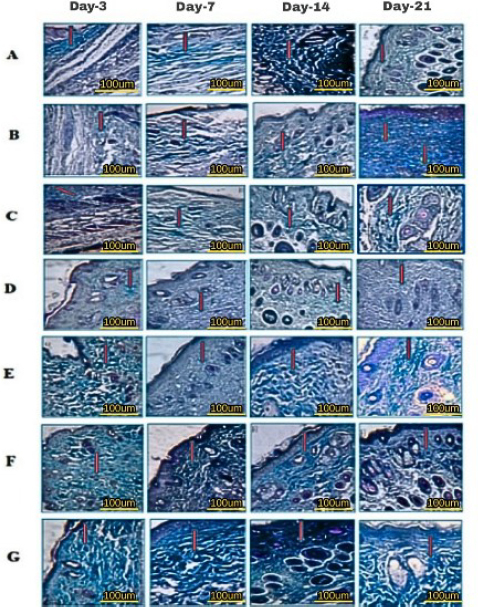
Hydrogel treatment for collagen formation red arrow using Trichome Mallory staining on days 3, 7, 14 and 21; A - healthy untreated, B - DM untreated, C - DM treated with Carbopol, D - DM treated with commercial 15 %, E - DM treated with hAMD 15 %, F - DM treated with spidroin 15 % and G - DM treated with composite 10 %

**Figure 18.**
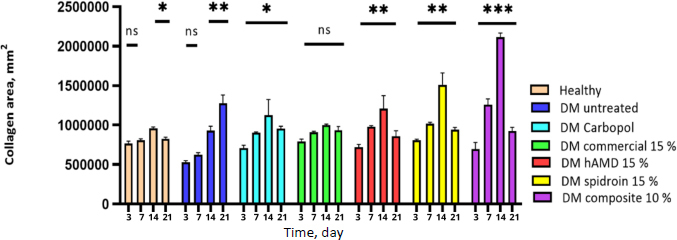
The results of the histogram of the amount of collagen showed there was an increase in the thickness of the blue colour in all groups from day 3 to day 7; from day 7 to day 14, there was an increase again in all groups and from day 14 to day 21 there was a decrease except for the DM group without treatment. The symbol *** indicates a significant difference (*p*<0.05), *n* = 4

The increase in collagen density observed from day 3 to day 14 was significant in all groups (**). In the 10 % composite group, there was a very high increase (***), and on day 21, all groups experienced a decrease except the untreated DM group. This increase in collagen indicates faster wound healing and improved structural integrity of the repaired tissue (Figure 17, histology; Figure 18, histogram).

**Figure 19.**
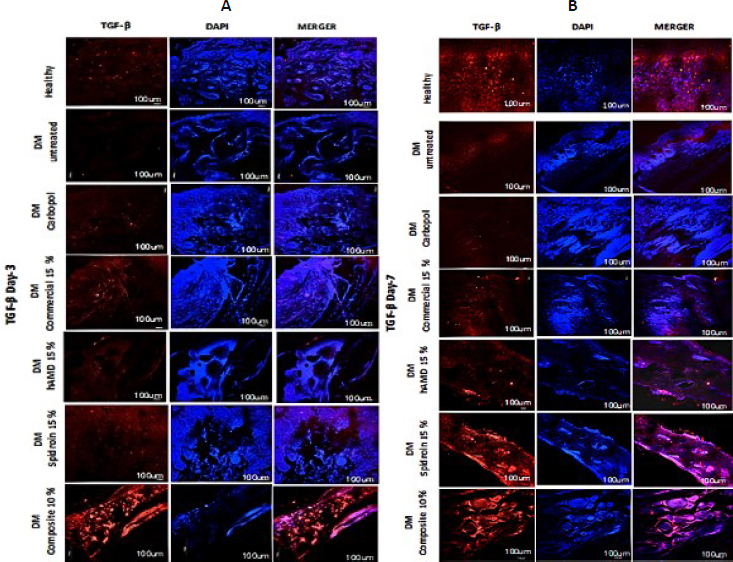
Hydrogel treatment of TGF-β expression using IHC staining. Red (TGF-β) and blue/DAPI (cell nuclei) using TGF-β primary antibody and secondary antibody (Alexa Fluor 647); A - day 3, B - day 7

**Figure 20.**
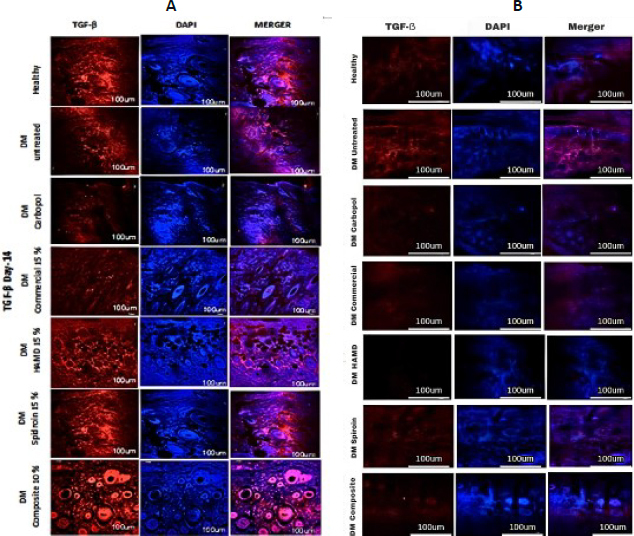
Hydrogel treatment of TGF-β expression using IHC staining. Red (TGF-β) and blue/DAPI (cell nuclei) using TGF-β primary antibody and secondary antibody (Alexa Fluor 647); A - day 14, B - day 21

**Figure 21.**
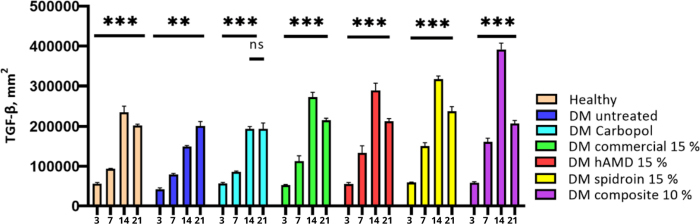
The results of the histogram of TGF-β expression using IHC staining, the symbol *** indicate a significant difference (*p*<0.05).

The increase in TGF observed from day 3 to day 14 occurred in all groups. In the 10 % composite group, there was a significant increase (***), and on day 21, all groups experienced a decrease except the untreated DM group. Reducing transforming growth factor beta (TGF-β) levels during the wound remodelling phase is an important mechanism to prevent excessive fibrosis and ensure optimal tissue maturation (Figures 19 and 20, histology; Figure 21, histogram).

Based on this study, HAM has functional groups at specific wavenumbers, such as 1652, 1550, 1339 and 1200 cm^-1^. This study showed that ATR-FTIR results of HAMD have functional groups exhibiting absorption bands at 1652, 1550 and 1339 cm^-1^ [31]. Furthermore, spidroin has functional groups at a specific wavenumber of 1530 cm^-1^. This study revealed that ATR-FTIR results of the spidroin *Argiope appensa* have a functional group of 1530 cm^-1^ [32]. ATR-FTIR confirms the presence of structural proteins in the amniotic membrane, such as collagen and laminin, which are essential for cell adhesion, epithelial cell migration, and stimulation of new blood vessel growth (angiogenesis). During wound closure, the amniotic membrane acts as a stimulator of new tissue growth, a reservoir of growth factors, and a barrier against infection and dehydration [33].

In this study, nine types of hydrogels were tested. Based on organoleptic and pH measurements, the hydrogel has met the criteria save for skin, including no odour, soft and flexible, transparent, and having a pH of around 5.8 and 6.1. The pH of human skin typically ranges from 4.5 to 6.0, with higher pH values occurring in intertriginous areas [34]. Maintaining an appropriate pH level in wound dressing can mitigate the potential for skin irritation [35]. The hydrogel was morphologically examined using SEM, revealing the presence of pores. Furthermore, the hydrogel has functional groups.

The swelling proportion is characterized as the incremental increase in the weight of the hydrogel due to water retention. The sol division speaks to the division of the polymer following a crosslinking response that's not a part of a crosslinked arrangement. A diminishing sol division over time reflects polymer failure and characterizes the degree of hydrogel degradation [36]. According to this study, the percentage of hAMD 15 % is significantly lower compared to Carbopol, commercial 15 %, spidroin 15 % and composite 10 %.

In the present study, the potential of hAMD and spidroin formulations to accelerate the wound closure process in diabetic mice was investigated. The results of this study indicated that a combination of hAMD and spidorin quickens the wound closure process. This study revealed that hydrogel composite 10 % exhibits an increased speed of wound closure compared to other hydrogel formulations; composite 10 % shows increased wound closure capabilities, specifically within a 14-day duration, as indicated by the analysis of wound area and morphology as compared to healthy mice and untreated DM. This study, in line with [37], showed that spidroin accelerated the healing of the skin of rats by 19 days compared with the control group. Ulcers of the limits treated with an amniotic layer appeared at a more prominent healing rate than routine dressing during 6 weeks [38]. This study, in line with [37], demonstrated that spidroin accelerated skin healing in rats by 19 days compared to the control group. Ulcers treated with an amniotic membrane layer showed a significantly higher healing rate than those treated with routine dressings over a period of six weeks [38]. The conceivable nearness of development variables in human amniotic membrane, including TGF-β, EGF, PLGF, bFGF, GCSF, and cytokines, including IL-4, IL-6, IL-8, and IL-10 [39].

The results of inflammatory cells in the untreated DM group were less than those in the healthy and treated groups. Uncontrolled glucose levels can change/decrease the function of immune cells. In line with research by [40], a decrease in the number of inflammatory cells in the inflammatory phase causes a reduction in the body's ability to respond to infection, resulting in a persistent inflammatory state, which can delay the healing process. Another possibility is that it can cause the inflammatory condition to worsen, which can result in deregulation of the healing process phase, expansion of tissue damage, oxidative stress, and repair time; this could trigger chronic wounds or cause excessive scarring. However, in the proliferation phase in this study, on day 14 in the untreated DM group, the number of inflammatory cells increased (Figure 10), suggesting the possibility of transition experiencing disruption due to dysregulation of cytokine signals and persistence of inflammatory mediators [41,42]. If inflammatory cells occur continuously in the remodeling stage, it can cause tissue damage characterized by the retention of pro-inflammatory macrophages [43,44]. This can cause chronic wounds and increase apoptosis of fibroblasts and endothelial cells, leading to impaired wound healing [45]. Previous research [43] showed that a reduction in macrophages early in the healing process resulted in a significant delay in wound closure. The number of macrophage cells and neutrophils in the inflammatory phase and the balance between pro-inflammatory and anti-inflammatory signals are critical for effective wound healing.

The amount of angiogenesis increased in all groups from the inflammatory stage, day 3, to the proliferation stage, day 7 (Figure 12). The increase in angiogenesis is due to the inflammatory phase, in which inflammatory cells release growth factors and cytokines to stimulate angiogenesis. Growth factors act as significant angiogenesis stimulators by inducing migration and proliferation of endothelial cells, increasing vascular permeability, and forming granulation tissue, fibroblasts, and collagen. In line with research by [46], hAMD is known to improve angiogenesis in diabetic wounds because growth factors can encourage neovascularization. Besides growth factors, hAMD has pro-angiogenic factors, such as VEGF, bFGF, TGF-β, cytokines, and angiogenin. The aim is to stimulate the proliferation and migration of endothelial cells, which are essential processes in forming new blood vessels [47,48], and delivering nutrients and oxygen to wound tissue.

This study found the highest fibroblasts in the 10 % composite treatment group (Figure 14). It is possible that the increase in fibroblasts occurred because the biological properties of hAMD facilitate the production of fibroblasts, which are rich in growth factors (FGF, VEGF, PDGF, and TGF-β), cytokines, and ECM components, which have an essential role in the process of tissue regeneration and repair. Apart from that, according to research by [39,49], hAMD, which contains a bioactive matrix, has anti-inflammatory properties, the ability to provide a supporting matrix for cellular activity, and can also increase the recruitment of mesenchymal progenitor cells to the wound site, so that it can accelerate the wound healing process. This is in line with research by [50]. HAMD can modulate inflammatory responses, reduce inflammation, and create an environment supporting fibroblast activity and tissue repair. Apart from hAM, spidroin can produce amino acids, an additional ingredient for tissue regeneration, and contributes to adhesion and cell proliferation on its surface [51,52]. Spidroin consists of 40 % glycine and 25 % alanine as the primary amino acids. This matter shows that the treatment group given a hydrogel spidroin net has an effect on increasing the number of fibroblast cells compared with a healthy control group, an untreated DM. This is possible because the alanine in the net spider hydrogel acts as an anti-inflammatory and can reduce the inflammatory response, so the healing process occurs more quickly [52,53], and the properties of this composite can heal wounds effectively.

A stronger understanding of epithelialization preparation may provide insights for novel therapeutic approaches to rapid wound closure [54]. hAMD and spidroin were superior in accelerating wound closure and epithelialization within a 14-day duration (Figure 16). HAMD stimulates epithelial cell migration and adhesion in skin wounds. Multiple growth factors are involved in the process, such as EGF, PLGF, TGF-β, and bFGF. Granulocyte colony-stimulating factors are among the growth factors that contribute to this (GCSF) [55]. The present study shows the beneficial interaction between hAMD and spidroin. Hydrogel composite with a concentration of 10 % shows considerable potential as an effective wound closure method, characterized by its effect on wound closure and histological results. This study shows a beneficial combination of hAMD and spidroin.

The increase in collagen density in all groups observed from day 3 to day 14 was significant (Figure 18). The 10% composite group showed the highest increase in collagen density. Increasing collagen density and making strides in the basic healing of repaired tissue not only quickens wound closure but can also anticipate complications, such as prolonged aggravation that can prevent ordinary healing. In line with the inquiry, about [55-57], collagen in hAM can speed up the healing of burns and wounds on the skin of individuals with diabetes. On day 21, all groups experienced a decrease in collagen density. The most significant reduction occurred in the 10 % composite group, so excessive scar tissue formation could also be minimized. In any case, collagen increment occurred once more within the untreated DM group on day 21. This increment was most likely caused by uncontrolled blood sugar levels or hyperglycemia, which disturbed the generation of TGF-β and VEGF, development components that are vital for wound healing [58].

TGF-β is essential in chronic wound healing because it alters re-epithelialization, inflammation, angiogenesis, and granulation tissue organization. TGF-β is transmitted by inflammatory cells, including macrophages, fibroblasts, keratinocytes, and platelets. TGF-β may be a multifunctional cytokine that plays a fundamental part in cell development, division, apoptosis, and resistance regulation. TGF-β is required in every organ of the constant skin wound that heals through several stages; it influences the inflammatory response [59], stimulates angiogenesis [60] and fibroblast expansion, produces collagen fusion, and enhances re-epithelialization. Thus, the higher the TGF-β level, the higher the multiplication of fibroblasts that depend on collagen fusion. In this contemplation, the solid group, untreated DM, and all treated DM from the Inflammation to the proliferative stage experienced a significant increase (Figure 20). However, in the untreated DM group, the amount of TGF-β was the smallest. It is most likely a combination of metabolic conditions, hyperglycemia and microcirculation that needs to activate the TGF-β pathway.

On the other hand, in the DM group treated with 10 % composite, the amount of TGF-β was the highest; spidroin may interact with specific cells to trigger TGF-β secretion. ECM, cytokines, and growth factors derived from hAM can act as chemoattractants for various types of immune cells. In the 10 % composite group at the remodelling stage (day 21), because the wound has healed, excessive TGF-β expression in myofibroblasts can be minimized to prevent the formation of hypertrophic scar tissue.

## Conclusions

This study demonstrates that wound healing in the group of diabetic mice treated with a 10 % composite (a combination of hAMD and spidroin) was significantly better and faster compared to the healthy control group, untreated DM group, DM groups treated with Carbopol, DM groups treated with 15 % commercial, DM groups treated with 15 % hAMD, and DM groups treated with 15 % spidroin. The enhanced healing is attributed to multiple mechanisms, including increased cell proliferation, reduced inflammatory cell infiltration, enhanced angiogenesis, fibroblast activity, re-epithelialization, collagen synthesis, and upregulation of TGF-β. This composite shows potential as an alternative treatment for diabetic wounds, not only promoting wound healing but also minimizing excessive scar tissue formation. However, further studies are required to investigate the underlying molecular pathways. Future research may also involve larger animal models such as guinea pigs or rabbits. These findings have the potential to inspire and motivate further research into chronic diabetic wound healing, ultimately contributing to significant advancements in the care and treatment of chronic wounds in diabetic patients.
